# *Ommexecha virens* (Thunberg, 1824) and *Descampsacris serrulatum* (Serville, 1831) (Orthoptera, Ommexechidae): karyotypes, constitutive heterochromatin and nucleolar organizing regions

**DOI:** 10.3897/compcytogen.v5i2.960

**Published:** 2011-06-01

**Authors:** D.B. Carvalho, M.F. Rocha, V. Loreto, A.E.B. Silva, M.J. Souza

**Affiliations:** 1 Departamento de Genética, Centro de Ciências Biológicas/CCB, Universidade Federal de Pernambuco/ UFPE, Av. Prof. Moraes Rego s/n, Recife, Pernambuco, Brasil CEP:50732-970; 2 Instituto de Ciências Biológicas/ICB, Universidade de Pernambuco /UPE, Recife, Pernambuco, Brasil; 3 Departamento de Botânica, Centro de Ciências Biológicas/CCB, Universidade Federal de Pernambuco/ UFPE

**Keywords:** FISH, grasshopper, Ommexechidae, rDNA, *Ommexecha virens*, *Descampsacris serrulatum*

## Abstract

Chromosomes of *Ommexecha virens* and *Descampsacris serrulatum* (Ommexechidae) were analyzed through conventional staining, C-banding, base specific fluorochromes, silver nitrate impregnation (AgNO3), and fluorescent in situ hybridization (FISH) with probe for 45S rDNA. The two species presented diploid number 2n= 23,X0 in males and acrocentric autosomes, except the pair one that presented submetacentric morphology. The X chromosome has distinct morphology in the two analyzed species, being a medium acrocentric in *Ommexecha virens* and large submetacentric in *Descampsacris serrulatum*. The C-banding revealed pericentromeric blocks of constitutive heterochromatin (CH) in all the chromosomes of *Descampsacris serrulatum*. For *Ommexecha virens* it was evidenced that the blocks of CH are preferentially located in the pericentromeric area (however some bivalents presents additional blocks) or in different positions. The staining with CMA3/DA/DAPI showed GC rich CH blocks (CMA3+) in some chromosomes of the two species. The nucleolar organizer regions (NORs) were located in the bivalents L2, S9, S10 of *Ommexecha virens* and M5, M6, M7, S11 of *Descampsacris serrulatum*. The FISH for rDNA showed coincident results with the pattern of active NORs revealed by AgNO3. This work presents the first chromosomal data, obtained through differential cytogenetics techniques in Ommexechidae, contributing to a better characterization of karyotypic evolution for this grasshopper family.

## Introduction

The family Ommexechidae is endemic to South America, constituted by two subfamilies (Aucacrinae and Ommexechinae) and includes about 12 genera and 30 species. *Descampsacris* is a monotypic genus represented by *Descampsacris serrulatum*. On the other hand, the genus *Ommexecha* comprises seven species, *Ommexecha apolinari*, *Ommexecha brunneri*, *Ommexecha germari*, *Ommexecha giglio-tosi*, *Ommexecha gracilis*, *Ommexecha macropterum* and *Ommexecha virens*. This genus has a wide geographical distribution being found from the Andes to Caribbean. Among the described species, *Ommexecha virens* presents great morphologic and chromatic variability (Ronderos 1979).

Mesa et al. (1982) reported cytogenetic data on 13 Ommexechidae species, mainly with karyotype 2n= 23, X0. However, *Ommexecha germari* has the smallest chromosome number (2n= 21, X0) and *Conometopus sulcaticollis* showed 2n= 25, X0, the largest diploid number for the family. *Spathalium helios* demonstrates 2n = 22, neoXY with the X chromosome metacentric corresponding to the largest element of the karyotype and Y showed acrocentric morphology (Mesa et al. 1990). This species demonstrates also the unique representative of Ommexechidae analyzed by differential staining technique, showing constitutive hetrochromatin (CH) in the centromeric region of the autosomes and additional terminal blocks in the pairs seven and eight (Mesa et al. 1990).

Differential cytogenetic staining in grasshoppers of the families Acrididae and Romaleidae, from Neotropical Region, has shown a variability in distribution pattern and qualification of the CH through the C-banding and base specific fluorochromes Chromomycin A3 (CMA3) and 4’-6’-diamidino-2-fellindol (DAPI) staining. Distinct nucleolus organizing regions (NORs) locations, through the silver nitrate impregnation (AgNO3) and fluorescent *in situ* hybridization (FISH), have been observed in these species (Souza et al. 1998, Loreto and Souza 2000, Pereira and Souza 2000, Souza et al. 2003, Loreto et al. 2005, Souza and Melo 2007, Loreto et al. 2008).

In this work chromosomes of *Ommexecha virens* and *Descampsacris serrulatum* were analyzed through conventional staining, C-banding, base specific fluorochromes, impregnation with AgNO3 and FISH with probe of 45S rDNA. The karyotypic patterns obtained contributed to a better understanding about chromosomal evolution in the family Ommexechidae.

## Material and methods

In this work 36 males of *Ommexecha virens* (Thunberg, 1824) and 11 of *Descampsacris serrulatum* (Serville, 1831) were analyzed. The species studied were collected in different areas in the states of Pernambuco and Bahia in the Northeast Region of Brazil (Table 1). The testes were fixed in ethanol and acetic acid 3:1. The cytological preparations were obtained through squashing of testes follicles. For conventional analysis the slides were stained with lacto acetic orcein 2%. For the C-banding the technique of Sumner (1972) was used with small modifications in the time of use of the basic solution. In the sequence the slides were treated with HCl 0,2N by 25 minutes, barium hydroxide (Ba(OH)2) to 5% the (60°C) for 10 seconds and 2XSSC the (60°C) for 25 minutes. The slides were stained with 5% Giemsa during 5 minutes. The staining of CMA3/DA/DAPI was accomplished in agreement with Schweizer et al. (1983). After 5 days the slides were stained with CMA3 during 50 minutes, washed in distilled water and stained with Distamycin during 45 minutes, again washed and stained with DAPI for 20 minutes. The slides were mounted in glycerol/ Macllvaine buffer/MgCl2.

The silver nitrate impregnation (AgNO3) was done according to Howell and Black (1980). A 50% solution of silver nitrate was used (5g of AgNO3 in 10ml of distilled water), besides a colloidal solution (gelatin). The slides were incubated in humid camera at 70ºC during from 3 to 5 minutes.

Fluorescent *in situ* hybridization (FISH) was performed according to Moscone et al. (1996) using probe for 45S rDNA. The probe was labelledwith digoxigenin through nick translation and detected with the anti-digoxigenin sheep antibody linked to the fluorochrome FITC. A FITC-conjugated rabbit anti-sheep antibody (743, Dako) was used for amplification of hybridization signal. The chromosomal preparations were counterstained with DAPI (2µg/ml) and mounted in Vectashield H-1000 (Vector).

The cells submitted to the fluorochromes and FISH were captured through the image capture system Cytovision coupled to the microscope Olympus BX51. For the other methods the cells were photographed in microscope Leica, using a film Kodak Imagelink, Wing 25. The figures were mounted with the use of the program Corel Draw Graphics Suite 12 software.

**Table 1.** Species, localities of collections, geographical coordinates and number of analyzed individuals.

Species	Localities	Coordinates	No. of analyzed individuals
*Ommexecha virens*(Serville, 1831)	Buíque (PE)	8°37'23"S, 37°9'12"W	8
	Itamaracá (PE)	7°44'52"S, 34°51'19"W	9
	Sobradinho (BA)	9°27'19"S, 40°49'24"W	6
	Rio de Contas (BA)	13°34'44"S, 41°48'41"W	7
	Itaberaba (BA)	12°31'39"S, 40°18'25"W	6

*Descampsacris serrulatum* (Thunberg, 1824)	Rio de Contas (BA)	13°34'44"S, 41°48'41"W	6
	Andaraí (BA)	12°48'26"S, 41°19'53"W	3
	Mucugê (BA)	13°0'19"S, 41°22'45"W	2

## Results

**Figure 1.**
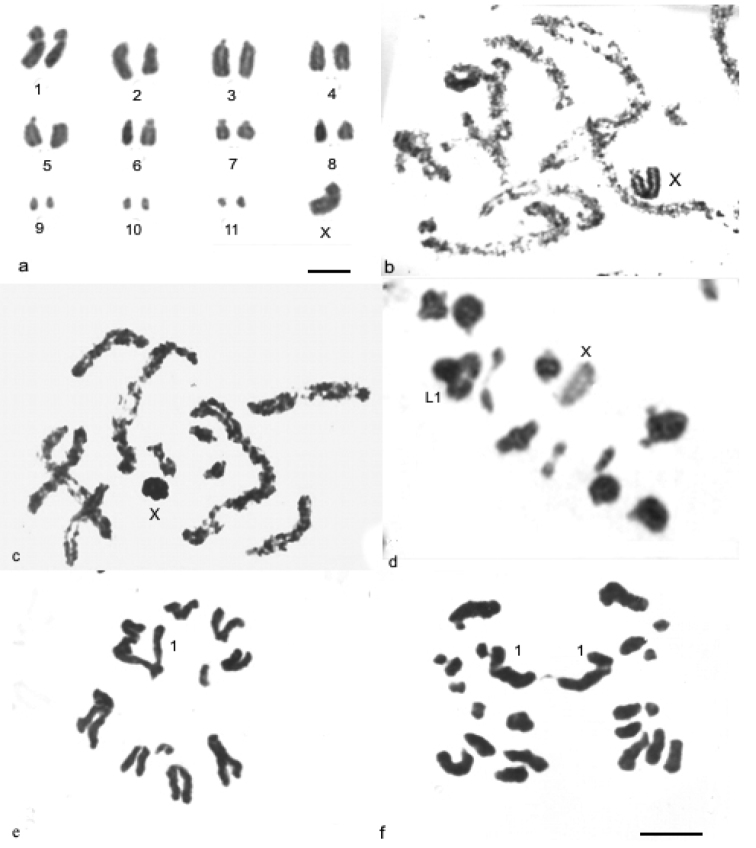
Conventional staining in mitotic and meiotic cells of *Descampsacris serrulatum* **a, b** and *Ommexecha virens* **c, f**. **a** spermatogonial metaphase **b** and **c** pachytene **d** metaphase I **e** metaphase II **f** anaphase II. Bar= 5µm.

*Descampsacris serrulatum* and *Ommexecha virens* demonstrate similar karyotypes with diploid number 2n= 23,X0 in the males and acrocentric autosomes, except the pair one that presented submetacentric morphology (Fig. 1, a-f). The autosomes were arranged according to their size as three large (L1-L3), five medium (M4-M8) and three small (S9-S11). *Descampsacris serrulatum* has one large X chromosome with submetacentric morphology (Fig. 1, a, b), while in *Ommexecha virens* the X is of medium size and acrocentric (Fig. 1, c, d). During the prophase I of the two species the X chromosome was heteropycnotic positive until the diplotene, being that behavior reverted in the metaphase I (Fig. 1, b-d).

**Figure 2.**
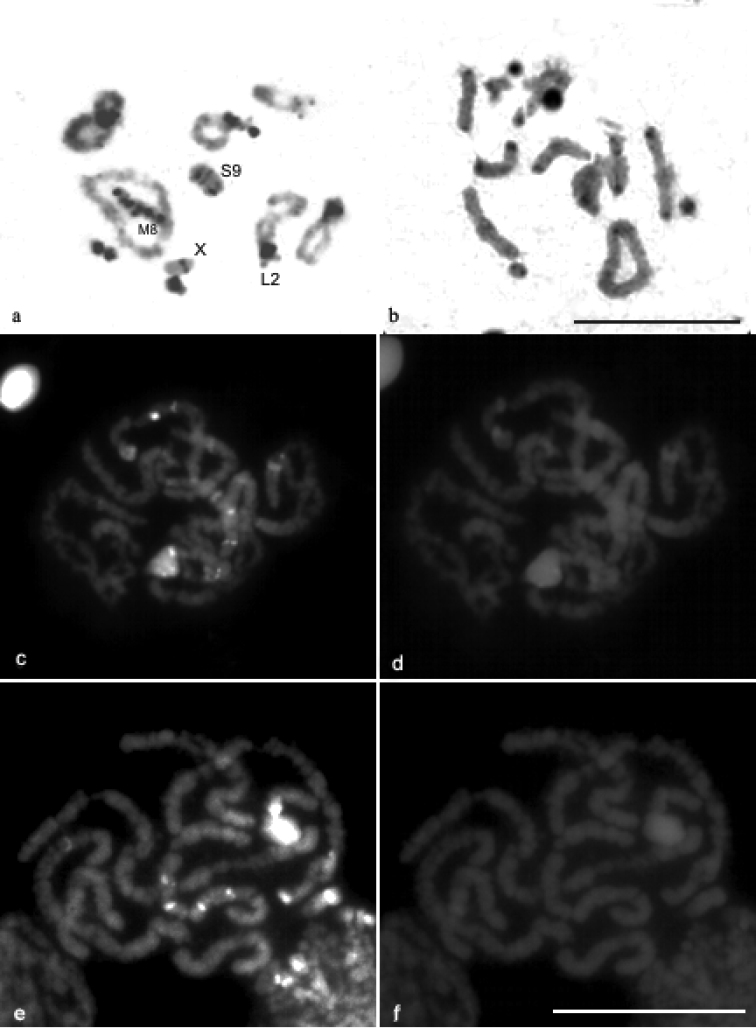
C-banding pattern in diplotene cells of *Ommexecha virens* **a** and *Descampsacris serrulatum* **b** Staining CMA3/DA/DAPI in pachytene cells of *Descampsacris serrulatum* **c** CMA3 **d** DAPI and *Ommexecha virens* **e** CMA3 **f** DAPI. Bar= 5 µm.

C-banding revealed distribution of constitutive heterochromatin (CH) in the pericentromeric region of all chromosomes of *Descampsacris serrulatum* (Fig. 2, b). In *Ommexecha virens* has blocks of CH preferentiality in the pericentromeric region, however some bivalents demonstrate additional blocks in different positions, such as L2 with proximal block, besides the pericentromeric; M8 showed intercalation of eu-heterochromatin along its extension; S9 presented a large block of CH extending from the pericentromeric to proximal region. Moreover the X chromosome showed proximal block (Fig. 2, a).

**Figure 3.**
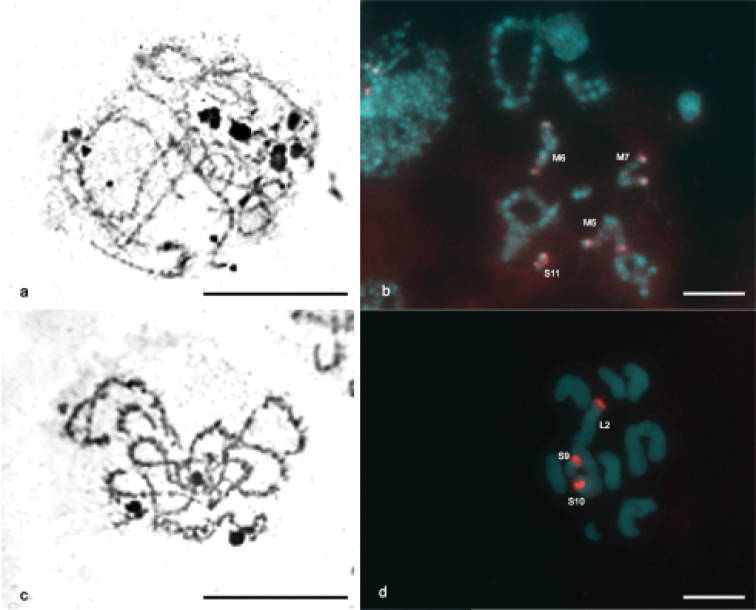
Impregnation with silver nitrate **a, c** and FISH with probe of 45S rDNA **b, d**. Zygotene and diakinesis of *Descampsacris serrulatum* **a, b** and zygotene and pachytene of *Ommexecha virens* **c, d**. In **b, d** are indicated the pairs containing the rDNA sites. Bar= 5 µm.

CMA3/DA/DAPI staining revealed blocks CMA3+ in some chromosomes of the karyotype of *Ommexecha virens*, as well as of *Descampsacris serrulatum* (Fig. 2, c, e). However, no AT positive blocks were detected in these two species (Fig. 2, d, f). Impregnation with AgNO3 identified nucleolus organizing regions (NORs) active in the two analyzed species. In *Descampsacris serrulatum*, NORs are located in four autosomal bivalents (M5, M6, M7 and S11) and in *Ommexecha virens* in three (L2, S9 and S10) (Fig. 3, a, c).

FISH with probe of 45S rDNA was used in meiotic cells of *Descampsacris serrulatum* and *Ommexecha virens* allowing the precise identification of rDNA sites observed by the impregnation AgNO3. In *Descampsacris serrulatum* it was observed that in four autosomal pairs the sites of rDNA are located in the pericentromeric regions (Fig. 3, b), besides the bivalent S11 presented a large sign of the hybridization, occupying about 2/3 of the chromosome length. In *Ommexecha virens* it was revealed that the bivalents L2 and S9 possess pericentromeric sites and the S10 proximal (Fig. 3, d).

## Discussion

In spite of the wide distribution of the Ommexechidae in South America, the cytogenetic studies in this family are scarce and based mainly on conventional method (Mesa et al. 1982). In the present study conventional chromosomal analysis showed that the two species analyzed have 2n= 23, X0 in the males with a remarkable large submetacentric bivalent (pair one). The occurence of a large autosomal submetacentric pair were frequently reported for Ommexechidae. This submetacentric pair probably originated from a pericentric inversion involving an original acrocentric chromosome of a common ancestor of the discussed species, representing a karyotypic marker for this family, occurring in 16 of the 19 species studied until now (Mesa and Ferreira 1977, Mesa et al. 1982, 1990). On the other hand *Clarazella bimaculata*, *Conometopus sulcaticollis* and *Pachyossa signata* demonstrate only acrocentric chromosomes in their karyotype. However, *Descampsacris serrulatum* and *Ommexecha virens* have autosomes with the same morphology. The first species shows the X chromosome with submetacentric morphology, while the second one has acrocentric X chromosome. The difference in the morphology of the X chromosome of the two species probably is a result of pericentric inversion from the ancestral acrocentric condition. In general, the karyotypes of *Ommexecha virens* and *Descampsacris serrulatum* coincide with karyotypes of most other cytogenetically studied species of Ommexechidae and with data of Mesa et al. (1982) although the X submetacentric of *Descampsacris serrulatum* has not been observedby these authors.

The pericentromeric pattern of distribution of constitutive heterochromatin observed in *Descampsacris serrulatum* and *Ommexecha virens* is quite common for the superfamíly Acridoidea. This pattern has also been described for several species of the Neotropical Region, belonging the families Acrididae and Romaleidae (Souza and Kido 1995, Rocha et al. 1997, Loreto and Souza 2000, Rocha et al. 2004, Loreto et al. 2005, Souza and Melo 2007). In Ommexechidae the only species, *Spathalium helios*, was studied with chromosomal C-banding technique until now. This species has pericentromeric blocks of CH, besides telomeric blocks in the pairs 7 and 8 (Mesa et al. 1990). For the two species analyzed in this work, significant differences were observed in some chromosomal pairs of *Ommexecha virens* (L2, M8, S9 and X) with CH in different positions or in larger amount than it was observed for the same chromosomal pairs of *Descampsacris serrulatum*. This difference can be attributed to amplification mechanisms or heterochromatin dispersion that are acting more intensely in *Ommexecha virens* than in *Descampsacris serrulatum*.

The pattern of qualification of CH visualized by the staining CMA3/DA/DAPI in *Descampsacris serrulatum* and *Ommexecha virens* showed blocks of positive CMA3 in some chromosomes of the karyotype. Similar patterns with the presence of CMA3+ blocks in some chromosomes were also described in *Belosacris coccineipes*, *Cornops aquaticum*, *Stenopola dorsalis*, *Stenacris xantochlorae* and *Tucayaca parvula* (Acrididae) (Loreto and Souza 2000, Rocha et al. 2004). In *Chromacris nuptialis* and *Chromacris speciosa* (Romaleidae) the CMA3+ blocks are restrict to one autosomal pair (Loreto et al. 2005). These patterns contrast with the described for *Xyleus angulatus*, *Phaeoparia megacephala* and *Xestotrachelus robustus* (Romaleidae) in which CH of all karyotypic complement demonstrates the richness for GC base pairs (Souza et al. 1998, Pereira and Souza 2000, Souza et al. 2003).

The references on the using of the AgNO3 impregnation and fluorescent *in situ* hybridization (FISH) for major rDNA show that in grasshoppers as a whole and in species belonging to the families Acrididae and Romaleidae, NORs are more frequently found in one or two autosomal bivalents (Rufas et al. 1985, Souza et al. 2003, Rocha et al. 2004, Souza and Melo 2007, Loreto et al. 2008). Variability in relation to NORs pattern location was observed in two analyzed species. *Ommexecha virens* showed active NORs in the pairs L2, S9, S10 and *Descampsacris serrulatum* demonstrated NORs involving four of autosomes pairs (M5-M7 and S11). The presence of NORs in four autosome pairs is unusual for grasshoppers, and have been described only in *Eyprepocnemis plorans*, *Heteracris litoralis* and *Gomphocerus sibiricus* (Rufas et al. 1985). Moreover, differential pattern of distribution of NORs was observed by Loreto et al. (2008) in the species belonging to the genus *Rhammatocerus*. *Rhammatocerus brasilensis* has three autosomal pairs bearing NORs and in contrast, *Rhammatocerus brunneri*, *Rhammatocerus palustris* and *Rhammatocerus pictus* showed NORs in a single autosomal pair.

The differential pattern of distribution of NORs observed in *Descampsacris serrulatum* and *Ommexecha virens*, could be explained by a probable amplification and dispersion of sites of rDNA (18S, 28S, 5.8S), leaving of an ancestral condition, in which a single autosomal pair would be NORs bearer. On the other hand, there is no coincident NORs bearing pairs among the two species and the possibility of different origins for the pattern of NORs in Ommexechidae can not be discarded.
